# Splenic Immune Response Is Down-Regulated in C57BL/6J Mice Fed Eicosapentaenoic Acid and Docosahexaenoic Acid Enriched High Fat Diet

**DOI:** 10.3390/nu9010050

**Published:** 2017-01-10

**Authors:** Nikul K. Soni, Alastair B. Ross, Nathalie Scheers, Otto I. Savolainen, Intawat Nookaew, Britt G. Gabrielsson, Ann-Sofie Sandberg

**Affiliations:** 1 Division of Food and Nutrition Science, Department of Biology and Biological Engineering, Chalmers University of Technology, SE-41296 Gothenburg, Sweden; alastair.ross@chalmers.se (A.B.R.); nathalie.scheers@chalmers.se (N.S.); otto.savolainen@chalmers.se (O.I.S.); brittg@chalmers.se (B.G.G.); ann-sofie.sandberg@chalmers.se (A.-S.S.); 2 Division of Systems and Synthetic Biology, Department of Biology and Biological Engineering, Chalmers University of Technology, SE-41296 Gothenburg, Sweden; intawat@chalmers.se or INookaew@uams.edu; 3 Department of Biomedical Informatics, College of Medicine, University of Arkansas for Medical Science, Little Rock, AR 72205, USA

**Keywords:** eicosapentaenoic acid (EPA)/docosahexaenoic acid (DHA), spleen transcriptomics, inflammation and immunity, NF-κB, arachidonic acid

## Abstract

Dietary *n*-3 fatty acids eicosapentaenoic acid (EPA) and docosahexaenoic acid (DHA) are associated with reduction of inflammation, although the mechanisms are poorly understood, especially how the spleen, as a secondary lymphoid organ, is involved. To investigate the effects of EPA and DHA on spleen gene expression, male C57BL/6J mice were fed high fat diets (HFD) differing in fatty acid composition, either based on corn oil (HFD-CO), or CO enriched with 2 g/100 g EPA and DHA (HFD-ED), for eight weeks. Spleen tissue was analyzed using transcriptomics and for fatty acids profiling. Biological processes (BPs) related to the immune response, including T-cell receptor signaling pathway, T-cell differentiation and co-stimulation, myeloid dendritic cell differentiation, antigen presentation and processing, and the toll like receptor pathway were downregulated by HFD-ED compared with control and HFD-CO. These findings were supported by the down-regulation of NF-κB in HFD-ED compared with HFD-CO fed mice. Lower phospholipid arachidonic acid levels in HFD-ED compared with HFD-CO, and control mice suggest attenuation of pathways via prostaglandins and leukotrienes. The HFD-ED also upregulated BPs related to erythropoiesis and hematopoiesis compared with control and HFD-CO fed mice. Our findings suggest that EPA and DHA down-regulate the splenic immune response induced by HFD-CO, supporting earlier work that the spleen is a target organ for the anti-inflammatory effects of these *n*-3 fatty acids.

## 1. Introduction

Inflammation is an important biological process for the maintenance of tissue homeostasis. However, sustained inflammation could lead to certain diseases [1]. Chronic low-grade inflammation and immune system activation are observed in abdominal obesity and may have a role in the pathogenesis of obesity related diseases such as type-2 diabetes (T2D) and cardiovascular diseases (CVD) [2,3], and therefore is one of the main threats to health with increased risk of mortality and morbidity [4]. The World Health Organization reports that chronic diseases kills more than 38 million people worldwide [5]. Thus, resolution of low-grade inflammation has become a therapeutic target for preventing CVD and may have wider application in the prevention of chronic diseases. 

The spleen is a secondary lymphoid organ, with the primary function to filtrate the blood from damaged or senescent erythrocytes and antigens. During filtration, blood is exposed to mature lymphocytes leading to the production of new immune cells and antibodies. The spleen contains high levels of specialized T-cells and B-cells [6], and is therefore a suitable choice, e.g., for studies of the immune response during chronic inflammation. Inflammation is characterized by increase in white blood cell count [7,8], pro-inflammatory cytokines [9], and chemokines [10]. Diets high in saturated fatty acids are thought to stimulate chronic low-grade inflammation [11], whereas replacing saturated fatty acids with unsaturated fatty acids reduces the risk of inflammation [12]. Cell culture, animal studies, human trials and epidemiological studies have shown the potential preventive effects of *n*-3 polyunsaturated fatty acids in reducing inflammation [13,14,15,16]. 

The main polyunsaturated *n*-3 fatty acids in marine oils, eicosapentaenoic acid (EPA) and docosahexaenoic acid (DHA), are widely used as supplements and are purported to have a wide range of effects. It is unclear how *n*-3 fatty acids may reduce risk of inflammation, though there have been many mechanistic studies on *n*-3 fatty acids [17]. Although several studies have investigated the effects of *n*-3 fatty acids in spleen or splenocytes [18,19,20], few studies have investigated the effects of *n*-3 fatty acids on the spleen transcriptome [21]. We recently found that the innate and adaptive immune system in spleen was down-regulated by menhaden fish oil [21], a mix of fatty acids and other lipophilic compounds, including EPA and DHA. To explore the effects and understand the underlying mechanisms of pure EPA and DHA on the splenic immune response, we used transcriptomics to measure the response in gene-expression in spleen from mice who were fed a high-fat diet based on corn oil, partly replaced by EPA and DHA. Direct effects of the difference in fat intake on spleen fatty acid composition were also measured.

## 2. Materials and Methods

### 2.1. Animals and Ethical Declaration

Six-week-old male C57BL/6J mice were purchased from the Harlan Laboratories (Envigo, Horst, The Netherlands). They were caged 5–6 per group with ad libitum access to food and water throughout the study and were acclimatized in temperature and humidity controlled environment with 12-h dark/light cycle for three-weeks at a certified animal facility in Gothenburg, Sweden [22]. After acclimatization, the mice were fed either a HFD or normal diets (see Diets for details) for 8 weeks thereafter. Upon termination with intraperitoneal sodium barbital injection followed by cervical dislocation, spleen was dissected, weighed and snap-frozen in liquid nitrogen and stored at −80 °C until further use. The animal study was approved by the Animal Ethical Committee at Gothenburg University (Approval # 253-2009).

### 2.2. Diet

During the three-week acclimatization period, the mice were fed a standard control chow (based on AIN93m) [23]. There were no measured differences in mice body weight at the start of study (Table 1), and their body weights were measured weekly during the 8-week intervention study. Two mice from the control group were removed due to unsocial behavior, leaving *n* = 10 mice in control and *n* = 12 mice in each of the two HFD groups. HFD were used to induce obesity in the mice. In the two HFD groups, the HFD were matched for macronutrient content. The HFD was prepared either with 5% (*w*/*w*) corn oil, while the EPA and DHA enriched HFD was prepared with 3% (*w*/*w*) corn oil and 2% (*w*/*w*) EPA and DHA triglycerides (EPAX AS Lysaker, Norway). The content of EPA and DHA in HFD-ED diet was 8 g/kg [24,25]. The control diet provided 24% energy (E%) as protein, 12 E% as fat and 65 E% as carbohydrates, whereas the two HFD contained 25 E% as protein, 32 E% as fat and 44 E% as carbohydrates (Table 1). The diets were prepared by Lantmännen AB (Kimstad, Sweden). Mice had ad libitum access to water and diet, and the diets were changed three times per week during the study.

### 2.3. Splenic Fatty Acid Profiling

Approximately 100 mg spleen tissue was weighed, freeze-dried and extracted using the Folch total lipid extraction method [26]. Internal phospholipids (C17:0) and triglyceride (C19:0) standards (Nu-Chek prep, Inc., Elysian, MN, USA) were added to the samples prior to the separation of neutral fatty acids, free fatty acids and phospholipids fractions using solid phase extraction. The fatty acids profiles in each fraction was quantified by gas chromatography mass spectroscopy (GC-MS) as previously described [27].

### 2.4. RNA Isolation, Quality Assurance and Microarray Analysis

Spleen from four different mice from each group was selected for total RNA isolation on the basis of their body weight, plasma triglyceride and plasma cholesterol levels. Total RNA was purified using the RNeasy^®^ Plus Universal Mini kit (Qiagen Nordic, Sollentuna, Sweden). RNA integrity was estimated using RNA 6000 Nano LabChip for Agilent 2100 Bioanalyzer (Agilent Technologies, Santa Clara, CA, USA). RNA was quantified by NanoDrop 2000c UV-Vis Spectrophotometer (Thermo Scientific, Wilmington, NC, USA).

Microarray experiments were run at the Swegene Centre for Integrative Biology at Lund University Genomics core facility. Briefly, total RNA was labeled and hybridized to MouseWG-6_V2.0 Expression BeadChip (MouseWG-6_V2.0_R3_11278593_A; Illumina, CA, USA) containing 45,281 transcripts. The BeadChip content is derived from the National Center for Biotechnology Information Reference Sequence (NCBI RefSeq) database (Build 36, Release 22). The chip is supplemented with probes derived from the Mouse Exonic Evidence Based Oligonucleotide (MEEBO) set as well as examples protein-coding sequences from RIKEN FANTOM2 database. The data (raw and normalized) are deposited in SOFT-format at Gene Expression Omnibus (GEO) database under the accession number GSE76622.

The fluorescence intensities (raw signals), for the mean intensities of arrays were extracted from illumina bead array files using GenomeStudio Gene Expression software (GSGX v1.9.0, Illumina, San Diego, CA, USA). The data were quantile normalized, and variance-stabilizing transformation (VST) was performed using the default setting of lumiExpresso function from lumi package [28]. Empirical Bayes method from the Linear Models for Microarray Data (limma) package was then applied to the signals to calculate moderated t- and F-statistics, log odds and differential expression for comparisons between the diets [29]. 

Platform for integrative analysis of omics data (Piano) Bioconductor package was used for gene-set enrichment analysis (GSEA) for functional inference [30]. Before implementing the gene-set enrichment function from Piano, gene-set collection (gsc) files were prepared for gene ontologies (GO) related to biological processes (BPs), molecular function (MF) and cellular components (CC) and the other gsc file contains subset for BPs related to immune system process (GO:0002376). Furthermore, canonical signaling and metabolic pathways from the KEGG database was used for pathway analysis. Pathview function from the Pathview package was used to visualize the data, which renders KEGG pathway based on the experimental results [31]. As spleen is considered an important immunological tissue, we ran Piano for both, global and immune related changes in spleen for all diet combinations. In both gene-set analyses, gene-sets with less than 10 or more than 500 genes were excluded. For gene-expression analysis, a Benjamini–Hochberg corrected *p*-value < 0.001 for at least one diet comparison was considered significant.

### 2.5. Quantitative Real-Time PCR (qRT-PCR)

RNA of genes that were identified as being of especial interested based on the transcriptomics analysis was quantified to confirm the transcriptomics findings. Briefly, total RNA was isolated from selected mice spleen tissue and 10 ng/μL of total RNA were used for cDNA synthesis using universal cDNA synthesis kit to make the final reaction volume up to 10 μL (ExiLERATE LNA™ qPCR, Exiqon Inc., Woburn, MA, USA). Then, 1 μL of cDNA solution was diluted with 79 μL of nuclease free water. From this reaction, 4 μL of diluted cDNA was used for the qRT-PCR using the PCR starter kit (ExiLERATE LNA™ qPCR, Exiqon). The plate was run on Bio-Rad CFX96 (Bio-Rad Laboratories Inc., Hercules, CA, USA) real time detection system for 40 cycles following the manufacturers protocol. The KiCqStart™ SYBR green primers used were as follows: (a) Adipoq: *for*—CCACTTTCTCCTCATTTCTG, *rev*—CTAGCTCTTCAGTTGTACTAAC; (b) Ltf: *for*—CAAAAGGATAGATTCCCCAAC, *rev*—GTAACTCCTCAAATACCGTGC; (c) Ccl19: *for*—TTCTTAATGAAGATGGCTGC, *rev*—CTTTGTTCTTGGCAGAAGAC; and (d) Ubc: *for*—GAGACGATGCAGATCTTTG, *rev*—ATGTTGTAGTCTGACAGGG. Data analyses were performed by comparing ΔΔ*C*t values exported from the CFX manager software (BIORAD), using *Ubiquitin* (*Ubc*) as the reference gene and expressing changes relative to the control group.

### 2.6. Statistical Analysis

Differences due to diet in the physiological parameters and fatty acid concentrations measured were tested using one-way Anova followed by post-hoc Tukey-Honest significant difference test (Tukey-HSD). A *p*-value < 0.05 was considered significant unless otherwise stated. Statistical and sequencing data were analyzed in R Studio interface (Version 0.99.902—© 2009–2016 RStudio Inc., Boston, MA, USA) and packages from Bioconductor. The data are presented as mean ± SEM.

## 3. Results

### 3.1. Physiological Changes in Mice—Effects of High Fat Diets Differing in Their Fat Composition

No difference was observed in absolute spleen weight between the diet groups, though the HFD-CO fed animals had a lower spleen to body weight ratio (17%) compared with HFD-ED (*p*-value = 0.02) (Table 2). After eight-week intervention, the body weight gain was slightly higher in HFD-CO fed mice compared with HFD-ED fed mice (Table 2). We have previously reported higher plasma triglycerides and lower hepatic total lipids in the HFD-ED fed mice compared with HFD-CO fed mice [22].

### 3.2. Spleen Fatty Acid Profiles

Neutral lipids: The amount of total neutral lipid was higher in HFD-CO fed mice compared with HFD-ED but the amounts did not differ between control and HFD-ED fed mice (Table 3). The amount of saturated fatty acid (SFA) C14:0 was higher in HFD-CO compared with HFD-ED fed mice. The amount of C20:3 *n*-6 was lower in HFD-ED fed mice compared with control diet fed mice. C20:4 *n*-6 (arachidonic acid) did not differ between any compared diet groups. As expected, the amount of C22:6 *n*-3 (DHA) was higher in HFD-ED fed mice compared with mice fed HFD-CO (Table 3), whereas no C20:5 *n*-3 (EPA) was detected in the neutral lipid fraction.

Free fatty acids: Total free fatty acids (FFA) did not significantly differ between groups (Table 3). The amounts of C18:1 *n*-9 and C20:3 *n*-6 were higher in HFD-CO fed mice compared with the mice fed HFD-ED, whereas they did not differ between control fed mice compared with either HFD. The amount of C22:6 *n*-3 (DHA) was higher in HFD-ED fed mice compared with both control and HFD-CO fed animals (Table 3).

Phospholipids: The total phospholipids amount was lower in the mice fed HFD-ED compared with either control or HFD-CO fed mice (Table 3), though *n*-3 PUFAs including C20:5 *n*-3 (EPA), C22:5 *n*-3 (DPA) and C22:6 *n*-3 (DHA) were higher in HFD-ED fed mice. The amount of C18:2 *n*-6 (linoleic acid) was higher in HFD-ED fed mice compared with either control or HFD-CO fed animals whereas the amount of C20:3 *n*-6 and C20:4 *n*-6 (arachidonic acid) was higher in HFD-CO fed mice compared with the mice fed HFD-ED. The amount of SFA C12:0 was higher in HFD-CO fed mice compared with the mice fed control diet but did not differ from HFD-ED fed mice. No differences were observed for SFA including C14:0, C16:0, and C18:0 between any of the diet groups (Table 3).

### 3.3. Global Gene Expression Analysis of the Spleen Transcriptome

Changes in splenic gene expression were most obvious in response to HFD-ED compared to control or HFD-CO diets (Supplementary Materials Figure S1a). Principle component analysis of the spleen transcriptome did not detect any outliers in the data (Supplementary Materials Figure S1b) indicating good analytical reproducibility. At a stringent cutoff of *p*_adj_-value < 0.001, there were 1372 differentially expressed genes (DEGs) in splenic RNA from mice fed HFD-ED and 285 DEGs in the RNA from mice fed HFD-CO compared with control diet. Furthermore, to scrutinize dietary effects on the spleen transcriptome, GSEA on biological processes (BP) without immune system processes (GO:0002376) was implemented. For assessment of regulated BPs, *p*-value < 10 × 10^9^ for at least one comparison of HFD-ED, HFD-CO and control was considered significant. At this *p*-value cutoff, there were 110 BPs identified as differentially expressed (Supplementary Materials Figure S1c). Furthermore, KEGG pathway analysis of the spleen transcriptome for the comparison of HFD-ED vs. HFD-CO effects in mice, revealed down-regulation of arachidonic acid and linoleic acid pathways. For the arachidonic acid pathway in particular, HFD-ED down-regulated genes belonging to the cytochrome P450 superfamily, including the cytochrome P450 family 4 subfamily f polypeptide 14 (*Cyp4f14*), prostaglandin I2 (prostacyclin) synthase (*Ptgis* or *Cyp8a1*), and phospholipase A2 group IB pancreas (Pla2g1b), compared to HFD-CO group. Moreover, the glutathione peroxidase 1 (Gpx1) gene was up-regulated in the mice spleen, after ingesting a HFD-ED compared with a HFD-CO (Supplementary Materials Figure S1d). Given the role of the spleen as a secondary lymphoid organ [32], we further investigated the effects of EPA and DHA on the regulation of immune related BPs.

### 3.4. Immune System Related Gene Expression in the Spleen

**Figure 1:**
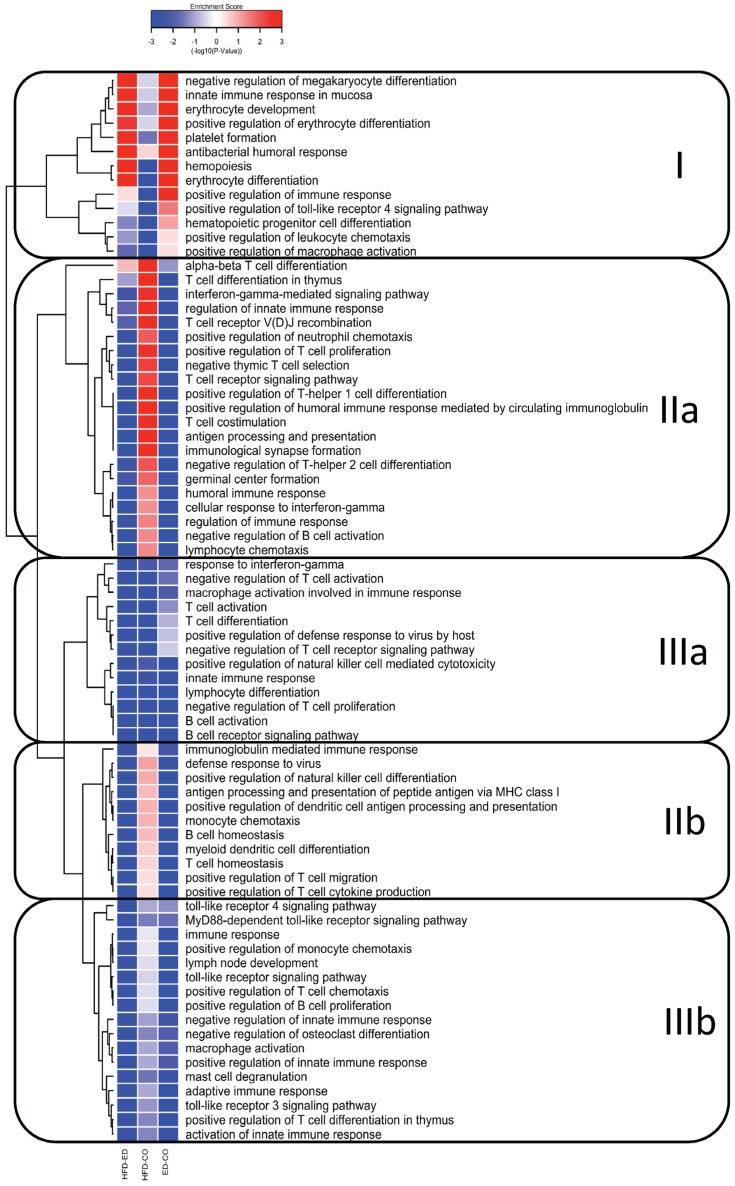
A hierarchical clustering and gene ontology (GO) enrichment analysis of DEGs showing 76 significantly regulated BPs (*p*-value < 0.001) related to immune system process for every diet comparison after eight weeks. The heat map is divided into four main clusters according to the regulation pattern and discussed in detail in results section. Color patterns indicate the direction of regulation, where red indicates up-regulation and blue indicates down-regulation of immune specific GO-terms. HFD-ED = HFD-ED vs. control diet; HFD-CO = HFD-corn oil vs. control diet; ED-CO = HFD-ED vs. HFD-CO.

To study the effect of including EPA and DHA in a HFD on immune system gene expression, we ran gene-set enrichment analysis on BP “immune system process” (GO:0002376). The resultant cluster analysis of 75 significantly different changes of immune related BPs (*p*-value < 0.001 in at least one comparison) can be classified into three clusters, which we have named I–III (Figure 1). Cluster I represent up-regulation of the BPs related to erythrocyte development and differentiation, hematopoiesis, negative regulation of megakaryocyte differentiation, and innate immune response in the mucosa, in the mice fed HFD-ED compared with control and HFD-CO, whereas these same BPs were down-regulated in the mice fed HFD-CO vs. control diet. Cluster IIa represents down-regulation of BPs related to both the innate and adaptive immune responses including T-cell signaling, T-helper cell 1 and 2 differentiation and T-cell co-stimulation, humoral immune response, interferon-gamma and an adaptive immune process such as α-β T cell differentiation in the mice fed HFD-ED compared with control and HFD-CO. Cluster IIb represents down-regulation of BPs related to T- and B-cell homeostasis, natural killer cell differentiation, antigen processing and presentation via MHC class I, and T-cell cytokine production by HFD-ED compared with the other two diets. These processes in cluster IIa and IIb were up-regulated by HFD-CO compared with control fed animals. Cluster IIIa represents down-regulation of BPs in both of the comparisons between HFD and the control diet. This included BPs mostly related to T- and B-cell activation and differentiation. Cluster IIIb represents pronounced down-regulation of BPs in the mice fed HFD-ED compared with HFD-CO, and included toll-like receptor 3 and receptor 4 signaling pathway, MyD88-dependent toll-like receptor signaling pathway, adaptive and innate immune response. These processes were also down-regulated by HFD-CO compared with control fed mice but were less pronounced compared to HFD-ED (Figure 1).

### 3.5. Modulation of NF-κB Related Gene Expression

**Figure 2:**
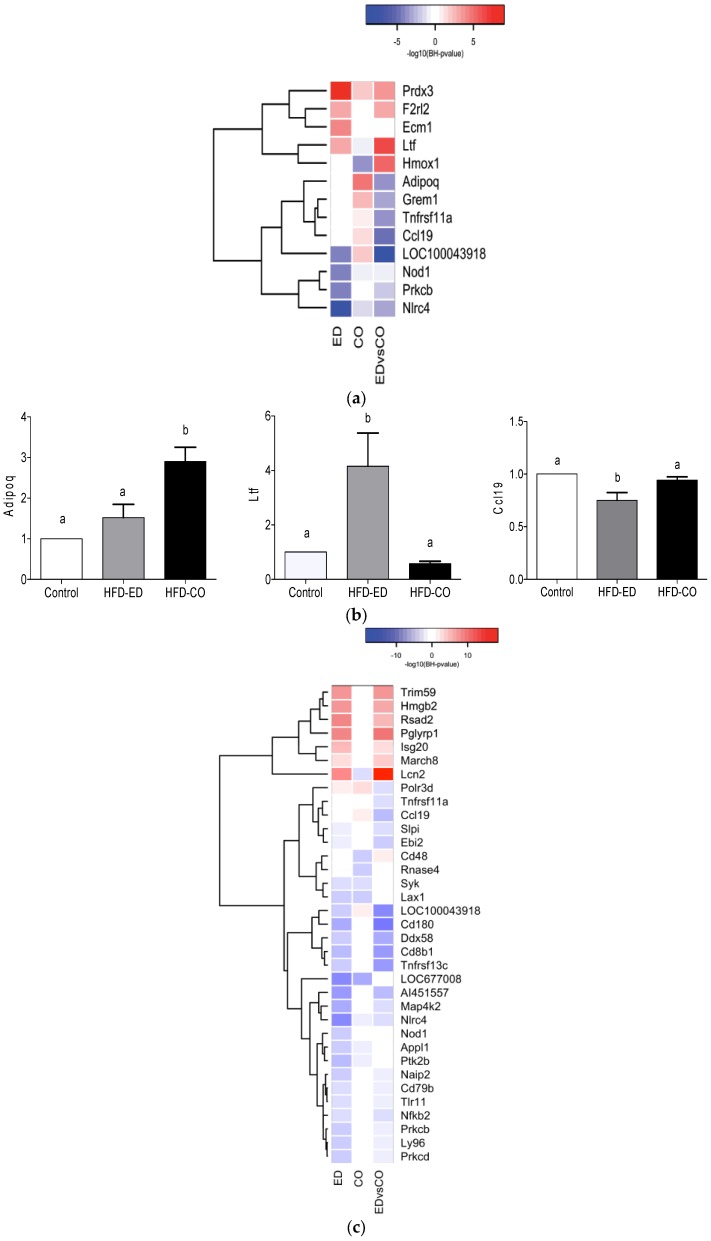

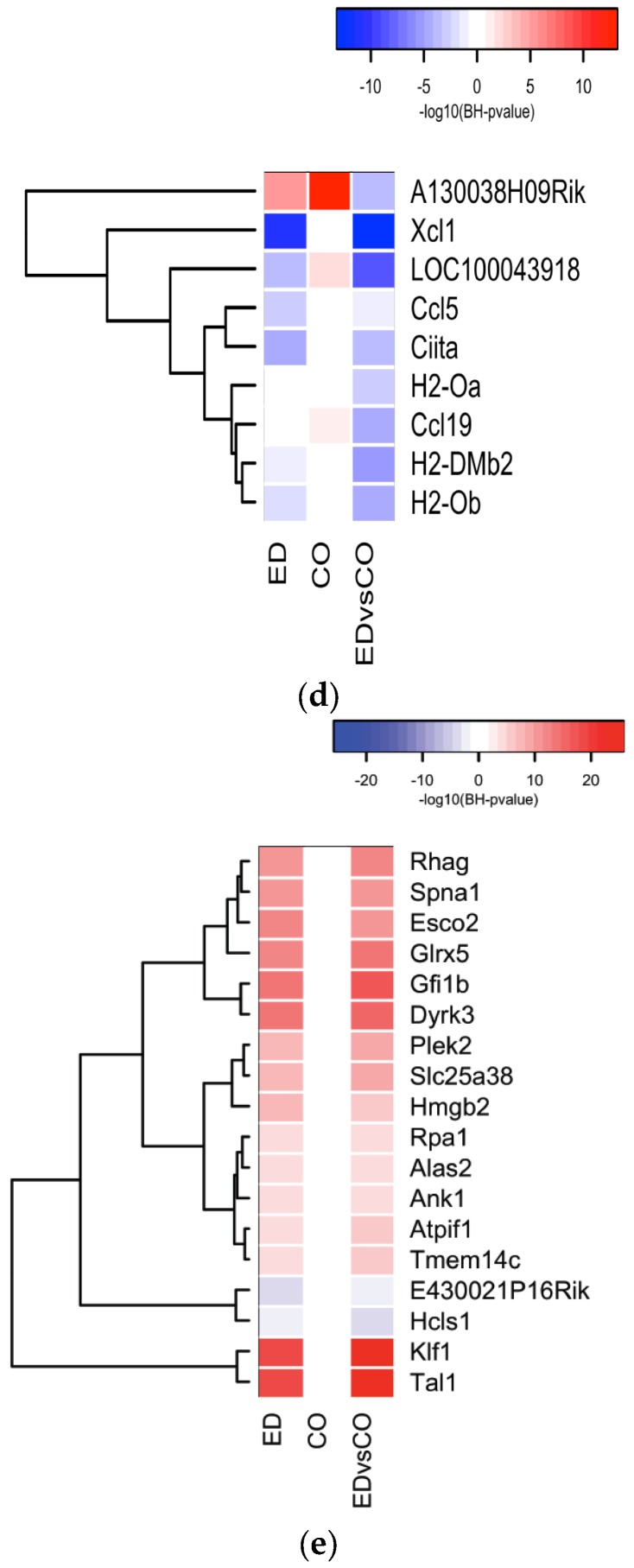
Heat map showing gene-expression patterns for any diet comparison i.e., HFD-ED vs. control; HFD-CO vs. control; HFD-ED vs. HFD-CO. for atleast one comparison BH-*p* value < 0.001 was considered significant. (**a**) Heat map showing group of genes regulated in NF-κB regulation, in BP GO:0043123 positive regulation of I-κB kinase/NF-κB signaling, BP GO:0051092 positive regulation of NF-κB transcription factor activity, and BP GO:0042346 positive regulation of NF-κB import into nucleus; (**b**) Relative expression of different genes involved in modulation of NF-κB in mouse spleen. Each gene is normalized against the house-keeping gene Ubiquitin (*Ubc*), and expression level shown as relative to the control diet; (**c**) Heat map showing groups of genes regulated in immune-related processes, including BP GO:0045087 innate immune response, BP GO:0002250 adaptive immune response, BP GO:0006959 humoral immune response, BP GO:0016064 immunoglobulin mediated immune response, BP GO:0001771 immunological synapse formation, BP GO:0042116 macrophage activation, BP GO:0002224 toll-like receptor signaling pathway, and BP GO:0002755 MyD88-dependent toll-like receptor signaling pathway; (**d**) Heat map showing groups of genes regulated in interferon-gamma regulation, including BP GO:0060333 interferon gamma mediated signaling pathway, BP GO:0019882 antigen processing and presentation, and BP GO:0071346 cellular response to interferon-gamma; (**e**) Heat map showing groups of genes regulated in erythrocyte turnover, including BP GO:0030218erythrocyte differentiation, BP GO:0048821 erythrocyte development, BP GO:0045648 positive regulation of erythrocyte differentiation, BP GO:0030097 hemopoieses, and BP GO:0002244 hematopoietic progenitor cell differentiation.

As a broad range of BPs related to immunity were affected by HFD-ED, we hypothesized that expression of NF-κB, a transcription factor that appears to link chronic low-grade inflammation and immunity to insulin resistance [2] and may influence obesity and related disorders, was accordingly affected. We compared the genes involved in the modulation of NF-κB for all three diets (Figure 2a). HFD-ED down-regulated genes, in comparison to either control or HFD-CO, that were involved in activation of the NF-κB signaling pathway directly or indirectly including Adiponectin C1Q and collagen domain containing (*Adipoq*), Gremlin 1 DAN family BMP antagonist (*Grem1*), Tumor necrosis factor receptor superfamily, member 11a NF-κB activator (*Tnfrsf11a*), Chemokine (C-C motif) ligand 19 (*Ccl19*), Nucleotide-binding oligomerization domain containing 1 (*Nod1*) a NOD-like receptor family of pattern recognition receptor, Protein kinase C beta (*Prkcb*), and NLR family CARD domain containing 4 (*Nlrc4*). The remaining 5 genes were up-regulated by HFD-ED compared with either control or HFD-CO and includes Peroxiredoxin 3 (*Prdx3*), Coagulation factor II (thrombin) receptor-like 2 (*F2rl2*), Extracellular matrix protein 1 (*Ecm1*), Lactotransferrin (*Ltf*), Heme oxygenase 1 (*Hmox1*). Relative expression of mRNA involved directly and indirectly involved in modulation of NF-κB including *Adipoq*, *Ltf*, *Ccl19* and *Tnfrsf11a* (*p*-value = 0.059; data not shown) verifies our transcriptome data (Figure 2b).

To observe the effects of dietary response on regulation of genes involved in innate, humoral and adaptive immune response including Toll-like receptors (Tlrs), the expression of immune-related genes was determined. Among the genes down-regulated by HFD-ED compared with the other two diets were Toll-like receptor 11 (*Tlr11*), Nucleotide-binding oligomerization domain containing 1 (*Nod1*), Mitogen-activated protein kinase kinase kinase kinase 2 (*Map4k2*), Nuclear factor of kappa light polypeptide gene enhancer in B cells 2, p49/p100 (*Nfkb2*), PTK2 protein tyrosine kinase 2 β (*Ptk2b*), CD180 antigen (*Cd180*) (Figure 2c). Cytokines and chemokines involved in the regulation of interferon-gamma mediated inflammation including Chemokine (C motif) ligand 1 (*Xcl1*), Chemokine (C-C motif) ligand 5 (*Ccl5*), Chemokine (C-C motif) ligand 19 (*Ccl19*), Histocompatibility 2, O region α/β locus (H2-Oa; H2-Ob) and Histocompatibility 2 class II locus Mb2 (*H2-DMb2*) were down-regulated in the mice fed HFD-ED, compared with the other two diets, while no changes were seen in the mice fed HFD-CO compared with control (Figure 2d). Genes associated with erythrocyte differentiation and development, and hematopoiesis were up-regulated in the mice fed HFD-ED compared with either diet but no changes were observed in HFD-CO fed mice compared with the control animals (Figure 2e). Plasma erythrocyte concentrations were not measured due to limited sample volume, but it has previously been shown that these genes may reflect increased turnover of erythrocytes without changing erythrocyte concentration in blood [33].

## 4. Discussion

We have investigated changes in the transcriptome profiles of mouse spleen tissue after an eight-week dietary intervention with control, HFD-ED or HFD-CO diets, the HFD-ED diet differing from the HFD-CO diet by the replacement of 40% of the corn oil (2% out of 5% CO) with purified triglycerides of EPA and DHA. We found that the gene coding for the lipid mediators of both the innate and adaptive immune response were markedly downregulated by the HFD-ED diet. The extensive down-regulation of the innate and adaptive immune system in mice spleen found in this study is in agreement with our previous study of spleen tissues from HFD-fish oil (menhaden oil) fed mice, compared to mice fed HFD-lard [21]. Several other rodent studies using splenic T-cells [18,34,35,36] also suggesting that EPA and DHA are the components in fish oil responsible for this effect.

In the present study, the spleen transcriptome was extensively influenced by EPA and DHA in the mice fed HFD-ED compared with the mice fed HFD-CO or control diet. Immune system related BPs including T- and B-cell activation and NF-κB related processes were down-regulated in the mice fed HFD-ED compared with either diets suggesting anti-inflammatory effects of a relatively low level of EPA and DHA enrichment of the diet. Contrary to our results, some other studies have found that B-cell response is enhanced in obesity when exposed to EPA and DHA [20,37]. These studies have been conducted in isolated murine B-cells, which may explain the divergent results. Moreover, BPs related to erythropoiesis were up-regulated in mice fed HFD-ED compared with either diets despite similar fat content, indicating possible increased turnover of red blood cells. Again, these data are supported by the previous study of spleen tissues from mice fed HFD-fish oil (menhaden oil) compared to HFD-lard [21]. 

Diets high in saturated fatty acids may contribute to greater serum levels of inflammatory mediators [11], though these results vary widely in humans [38,39]. Substituting saturated for unsaturated fatty acids can reduce the risk of inflammation [21]. However, the mechanism by which EPA and DHA impact the immune system and especially in spleen is not fully understood. Several hypotheses for how marine fatty acids, EPA and DHA in particular, may be protective against inflammatory disease have been proposed. These include alteration of cell membranes [40], modulation of NF-κB suppression [41,42], and altering of lipid rafts involved in T-lymphocyte signaling in immunological synapses [19]. A role for EPA and DHA in modulating the spleen-inflammatory response is well established [18,19,20,43], and our transcriptomics results support this role. Moreover, EPA and DHA derived resolvins, the E-series and D-series have chemically unique structural forms. A member of E-series, namely resolvin E1 reduces inflammation in vivo, and blocks human neutrophil transendothelial migration [44]. The DHA derived D-series resolvins, 17S and 17R D-series, are produced during the resolution of inflammation [45]. Thus, the difference in lipid mediators formed from the different fatty acid compositions in the diet may play an important role in effects on the spleen.

NF-κB is a transcription factor that regulates transcription of pro-inflammatory cytokines such as interleukin 6 and 12, interferon-gamma, and tumor necrosis factor [17,46]. Our study shows that HFD-ED can down-regulate certain tumor necrosis factors, as well as toll-like and nod-like receptors that are involved in activation of NF-κB transcription factor compared with the HFD-CO and control diets (Figure 2a,b). In addition, HFD-ED up-regulates the gene for peroxiredoxin 3 (*Prdx3*) involved in mitochondrial homeostasis [47]. This gene has been shown to be up-regulated in adipocyte oxidative stress, mitochondrial biogenesis and adipokines expression [48]. 

In addition, we found a pronounced decrease in the amount of phospholipid arachidonic acid in the spleen of HFD-ED mice compared to both HFD-CO and the control animals, showing down-regulation of the arachidonic acid pathway leading to the production of lipid mediators [49]. Lipid mediators from arachidonic acid have many functions, and can be both inflammatory and anti-inflammatory [49]. Due to insufficient tissue, further measurement of these lipid mediators was not conducted and should be measured in future studies to confirm this finding, and nor were there measurements of inflammation markers which would have indicated if the effect on phospholipid arachidonic acid concentrations had any effect on inflammation [49]. Linoleic acid, a precursor of arachidonic acid, was nearly two fold higher in the spleen phospholipids of HFD-ED fed mice compared to the other two groups, despite higher amounts of linoleic acid in the HFD-CO diets. One reason behind higher linoleic acid could be due to the fact that, dietary n-3 fatty acids down regulates the gene coding for desaturase [22,25,50], or another reason could be that linoleic acid competes with alpha linolenic acid for the same enzyme system for desaturation and elongation, and is likely that alpha linolenic acid would be preferentially desaturated and elongated to EPA and DHA [50]. However, the corn oil contains quite low-amounts of alpha linolenic acid, which make the latter suggestion less likely. Furthermore, we observed that HFD-ED down-regulated different chemokines such as Xcl1, Ccl5, and Ccl19 when compared with control or HFD-CO (Figure 2c). Disturbed cytokine and chemokine levels may lead to elevated inflammatory conditions [51]. Increased accumulation of the chemokines chemokine (C motif) ligand 1 (*Xcl1*) [52], chemokine (C-C motif) ligand 5 (*Ccl5*), and chemokine (C-C motif) ligand 19 (*Ccl19*) have been observed in infection [53]. Why the dietary enrichment with EPA and DHA has this effect is not clear, and we presume that the effect on phospholipid fatty acid composition may be in part responsible. 

HFD-ED down-regulated mitogen-activated protein kinase 2 (*Map4k2*) which is known to have similarity with other serine/threonine kinases found in human lymphoid tissue and are activated in stress response [54]. Different *Mapk* such as Extracellular signal-regulated kinase 1/2 (*Erk1*/*2*), C-Jun N-terminal kinase (Jnk) and p38 are upregulated in obesity-induced inflammation [55]. Moreover, down-regulation of Protein kinase C β, and δ (*Prkcb* and *Prkcd*) contributes to improved insulin resistance in HFD fed mice [56,57]. Our data show down-regulation of these protein kinases in HFD-ED fed animals compared with control but no changes were seen in HFD-CO compared with control fed animals suggesting that this may mediate lower HFD-induced insulin resistance in HFD-ED fed animals. Although circulating glucose and insulin concentrations were not measured in this study, liver fat and triglycerides were increased by 1.75 and 3 fold, respectively in the HFD-CO compared to HFD-ED [22]. Furthermore, Nuclear factor of κ light polypeptide gene enhancer in B cells 2, p49/p100 (*Nfkb2*) up-regulation is seen in inflammation and activation of the immune response [46], a gene that was down-regulated in HFD-ED fed mice. Nevertheless, a reduced immune response does not indicate a reduced capability to defend against bacterial infection. In a study using menhaden oil, Svahn et al. reported that this led to increased survival and decreased bacterial load in mice with septic infection, even though regulation of the splenic immune response was down-regulated [43].

By studying the effects of EPA and DHA on spleen, we have gained global insight that a relatively small change in dietary fat may have profound effects on spleen metabolism in the context of a HFD. Given the important role of the spleen as a secondary lymphoid organ in immune function and signaling, further research needs to determine if the observed downregulation of immune system related genes has an effect on other tissues, especially those that are known to be adversely affected by inflammation such as adipose tissue or vice-versa and most importantly, if the effects can be translated into humans. The amount of EPA and DHA fed to the mice was about 377 mg EPA and 577 mg DHA/kg per day, respectively. When converted to an equivalent dose in humans [58,59,60] approximately 9.5 mg/kg and 25 mg/kg was fed. This is somewhat higher relative to what a human would expect to get from eating fatty fish and similar to eating 3 g of fish oil capsules, suggesting that based on dose alone, these results may have relevance for humans. Future dose response studies are needed to establish whether the effects on transcriptomics and fatty acids are reproducible across a wider range of doses, and studies looking at the transcriptome effects across different ages are needed to establish whether long-term supplementation is required to observe the changes in gene-expression. Replication studies in humans are not feasible due to the need to get spleen biopsies from healthy subjects, though measuring work on immune markers and lipid meditators related to our findings may help to confirm that the spleen is affected by supplementation with EPA and DHA.

## 5. Conclusions

Overall, our data provide a novel insight into the regulation of the splenic transcriptome by EPA and DHA suggesting that EPA and DHA enriched corn oil down-regulate the splenic immune response induced by HFD. EPA and DHA also upregulated hematopoiesis. Furthermore, EPA and DHA decreased splenic phospholipid arachidonic acid concentrations, which could lead to reduced production of inflammatory mediators such as prostaglandins and leukotrienes.

## Figures and Tables

**Table 1:** The composition of the different diets used in this study. Data are adapted from [22]. Control = control chow; HFD-ED = high fat diet—eicosapentaenoic acid and docosahexaenoic acid; HFD-CO = high fat diet—corn oil.

Ingredient (g/100 g Diet)		Control	HFD-ED	HFD-CO
Protein	Casein	22.20	25.60	25.60
Carbohydrates	Sucrose	5.00	10.00	10.00
	Corn starch	56.00	34.80	34.80
	Cellulose	5.00	5.80	5.80
Fat	Total	5.00	15.00	15.00
	Corn oil	2.50	3.00	5.00
	Coconut oil	2.50	10.00	10.00
	EPAX oils ^a^	0.00	2.00	0.00
Minerals ^b^		2.00	2.50	2.50
Miconutrients ^c^		3.00	3.00	3.00
Choline bitartrate		1.60	2.00	2.00
Cholesterol		0.00	1.00	1.00
Methionine		0.20	0.30	0.30
Energy content (kJ/100 g)		1599	1752	1752
	Protein E%	24	25	25
	Carbohydrate E%	65	44	44
	Fat E%	12	32	32
Fatty acid composition ^d^ (mg/g diet)				
	C10:0	0.20	1.47	1.33
	C12:0	2.37	7.58	7.72
	C14:0	1.54	4.58	4.78
	C16:0	1.90	3.44	3.59
	C18:0	0.68	2.26	2.49
	SFA	6.70	19.33	19.91
	C18:1 *n*-9	2.82	4.80	5.26
	MUFA	2.82	4.80	5.26
	C18:2 *n*-6	3.62	5.03	7.36
	C18:3 *n*-6	0.12	0.22	0.26
	Total *n*-6 PUFA	3.74	5.26	7.62
	C20:5 *n*-3 (EPA)	0.00	2.03	0.01
	C22:6 *n*-3 (DHA)	0.00	4.58	0.01
	Total *n*-3 PUFA	0.00	6.61	0.02

^a^ EPAX 1050. EPAX 6015. ^b^ CaCO_3_ (57.7%); KCl (19.9%); KH_2_PO_4_ (11.9%); MgSO_4_ (10.4%). ^c^ Corn starch (98.22%); Ca(IO_3_)_2_ (0.0007%); CoCO_3_ (0.064%); CuO (0.02%); FeSO_4_ (0.5%); MnO_2_ (0.035%); Na_2_MoO_4_ (0.001%); NaSeO_3_ (0.0007%); ZnO (0.1%); Vitamin A (0.013%); B_2_ (Riboflavin-5-phosphate sodium; 0.027%); B_3_ (0.1%); B_5_ (Ca Pantothenate; 0.057%); B_6_ (0.023%); B_7_ (0.0007%); B_9_ (0.007%); B_12_ (0.00008%); D_3_ (0.007%); E (0.25%); K (0.003%). ^d^ Diet analyses were performed in triplicates, and the data were obtained by Gas chromatography mass spectroscopy.

**Table 2:** Changes in body weight composition and plasma lipid composition.

Parameter	Control	HFD-ED	HFD-CO
Total number of animals; *n*	9	12	12
Initial body weight (g)	27.50 ± 0.80	28.60 ± 0.80	24.30 ± 0.50
Final body weight (g)	31.40 ± 1.00	33.80 ± 0.80	36.20 ± 0.90
Change in body weight (g)	3.90 ± 0.50 ^a^	5.20 ± 0.30 ^a^	8.60 ± 0.50 ^b^
Absolute spleen weight (g)	0.09 ± 0.01	0.10 ± 0.00	0.09 ± 0.00
Spleen/body weight ratio (g/100 g)	0.29 ± 0.02 ^a,b^	0.29 ± 0.01 ^a^	0.24 ± 0.01 ^b^

The data are shown as mean ± SEM; different letters show significant different tested by ANOVA followed by Tukey’s multiple comparison test. To calculate the changes in body weight (g), initial body weight values from individual animals was subtracted from the final body weight measurement.

**Table 3:** The fatty acid profiles of different lipid fractions in spleen tissues (mg/g dry spleen biomass).

Neutral Lipids	Control *n* = 5	HFD-ED *n* = 8	HFD-CO *n* = 8
C12:0	0.00 ± 0.00	0.01 ± 0.00	0.06 ± 0.02
C14:0	0.01 ± 0.00	0.01 ± 0.00 ^a^	0.14 ± 0.06 ^b^
C16:0	1.03 ± 0.20	1.46 ± 0.22	3.34 ± 1.05
C18:0	0.21 ± 0.02	0.19 ± 0.04	0.13 ± 0.03
C18:1 *n*-9	1.78 ± 0.46	1.05 ± 0.17	1.43 ± 0.33
C18:2 *n*-6	0.03 ± 0.02 ^a^	0.2 ± 0.08 ^a^	0.86 ± 0.25 ^b^
C20:3 *n*-6	0.11 ± 0.01 ^a^	0.03 ± 0.01 ^b^	0.05 ± 0.02
C20:4 *n*-6	0.00 ± 0.00	0.00 ± 0.00	0.24 ± 0.14
C22:6 *n*-3	0.00 ± 0.00 ^a^	0.16 ± 0.04 ^b^	0.00 ± 0.00 ^a^
Total	3.16 ± 0.58	3.12 ± 0.43	6.25 ± 1.37
Free fatty acids			
C12:0	0.01 ± 0.00 ^a^	0.02 ± 0.00	0.04 ± 0.01 ^b^
C14:0	0.01 ± 0.00	0.02 ± 0.00	0.05 ± 0.01
C16:0	0.20 ± 0.02	0.20 ± 0.01	0.35 ± 0.07
C18:1 *n*-9	0.05 ± 0.00	0.05 ± 0.00 ^a^	0.06 ± 0.00 ^b^
C18:3 *n*-3	0.02 ± 0.00	0.02 ± 0.00	0.03 ± 0.00
C20:3 *n*-6	0.06 ± 0.01	0.02 ± 0.00 ^a^	0.07 ± 0.01 ^b^
C22:6 *n*-3	0.00 ± 0.00 ^a^	0.05 ± 0.00 ^b^	0.00 ± 0.00 ^a^
Total	0.35 ± 0.03	0.38 ± 0.02	0.60 ± 0.11
Phospholipids			
C12:0	0.00 ± 0.00 ^a^	0.01 ± 0.00	0.01 ± 0.00 ^b^
C14:0	0.07 ± 0.00 ^a^	0.15 ± 0.01 ^b^	0.13 ± 0.00 ^c^
C16:0	1.69 ± 0.05	1.70 ± 0.03	1.69 ± 0.05
C18:0	0.61 ± 0.02	0.59 ± 0.01	0.67 ± 0.04
C18:1 *n*-9	0.32 ± 0.01 ^a^	0.28 ± 0.01 ^b^	0.31 ± 0.01 ^a^
C18:2 *n*-6	0.12 ± 0.00 ^a^	0.22 ± 0.02 ^b^	0.14 ± 0.01 ^a^
C20:0	0.00 ± 0.00	0.01 ± 0.00	0.00 ± 0.00
C20:1 *n*-7	0.02 ± 0.00	0.02 ± 0.00	0.02 ± 0.00
C20:2 *n*-6	0.02 ± 0.00 ^a^	0.05 ± 0.01 ^b^	0.03 ± 0.00 ^a^
C20:3 *n*-6	0.17 ± 0.01 ^a^	0.18 ± 0.01 ^a^	0.25 ± 0.02 ^b^
C20:4 *n*-6	4.41 ± 0.10 ^a^	1.92 ± 0.05 ^b^	4.33 ± 0.08 ^a^
C20:5 *n*-3	0.00 ± 0.00 ^a^	0.41 ± 0.01 ^b^	0.00 ± 0.00 ^a^
C22:0	0.02 ± 0.01	0.02 ± 0.00	0.01 ± 0.00
C22:5 *n*-3	0.00 ± 0.00 ^a^	0.34 ± 0.01 ^b^	0.00 ± 0.00 ^a^
C22:6 *n*-3	0.20 ± 0.02 ^a^	0.82 ± 0.02 ^b^	0.19 ± 0.01 ^a^
Total	7.67 ± 0.19 ^a^	6.71 ± 0.13 ^b^	7.78 ± 0.22 ^a^

The fatty acid profiles from the mice fed either control, HFD-ED or HFD-CO are shown as mean ± SEM and as a proportion of total fatty acid fraction; different letters show statistical difference tested by ANOVA followed by Tukey’s multiple comparison test. For details see methods section.

## Supplementary Materials

The following are available online at http://www.mdpi.com/2072-6643/9/1/50/s1, Figure S1: (a) Venn diagram depicting overlaps among differentially expressed genes upon dietary intervention. Most regulation can be seen for comparison HFD-ED vs. control diet (ED vs. CD), and HFD-ED vs. HFD-corn oil (ED vs. CO). HFD-corn oil vs. control diet (CO vs. CD) shows comparatively less bidirectional differentially regulated genes; (b) Principle Component Analysis (PCA) plot based on the normalized gene expression from the spleen tissue fed control, HFD-ED and HFD-corn oil is plotted for assessing the quality of the datasets. No animals or any related data was excluded from further assessment; (c) A heatmap showing significantly regulated BPs (*p*-value < 10 × 10^9^) without immune system process for every diet comparison after 8 weeks. Changes in the diet, especially HFD-ED positively regulate BPs mostly related to DNA repair and cell cycle down-regulates NF-κB, interleukin-12 and interferon-gamma related processes. HFD-ED = HFD-ED vs. control diet; HFD-CO = HFD-corn oil vs. control diet; ED-CO = HFD-ED vs. HFD-corn oil; (d) Illustration of linoleic acid and arachidonic acid signaling pathway analysis of the splenic transcriptome for the comparison HFD-ED vs. HFD-CO fed mice. The genes highlighted in red including *Gpx1* (glutathione peroxidase 1) and *Alox12* (arachidonate 12-lipoxigenase) are up-regulated and the genes in green including *Pla2g1b* (phospholipase A2 group IB), *Cyp4f14* (cytochrome P450 family 4 subfamily f polypeptide 14) and *Ptgis* (prostaglandin 12 synthase) are down-regulated. In black are the lipid mediators and synthetic intermediates including prostaglandins, prostacyclins, leukotrienes, 5-oxo-eicosatetraenoic acid, 15-oxo-eicosatetraenoic acid, 12-hydroxy-eicosatetraenoic acid, 20-hydroxy-eicosatetraenoic acid.
