# Supplementary material for: Splenic Immune Response Is Down-Regulated in C57BL/6J Mice Fed Eicosapentaenoic Acid and Docosahexaenoic Acid Enriched High Fat Diet
Source: Nutrients. 2017 Jan 10;9(1):50. doi: 10.3390/nu9010050 (PMC5295094; doi:10.3390/nu9010050)
Supplement: Supplementary file 1 [file nutrients-09-00050-s001.docx]

Supplementary Materials: Splenic Immune Response Is Down-Regulated in C57BL/6J Mice Fed Eicosapentaenoic Acid and Docosahexaenoic Acid Enriched High Fat Diet

Nikul K. Soni, Alastair B. Ross, Nathalie Scheers, Otto I. Savolainen, Intawat Nookaew,
Britt G. Gabrielsson and Ann-Sofie Sandberg

| 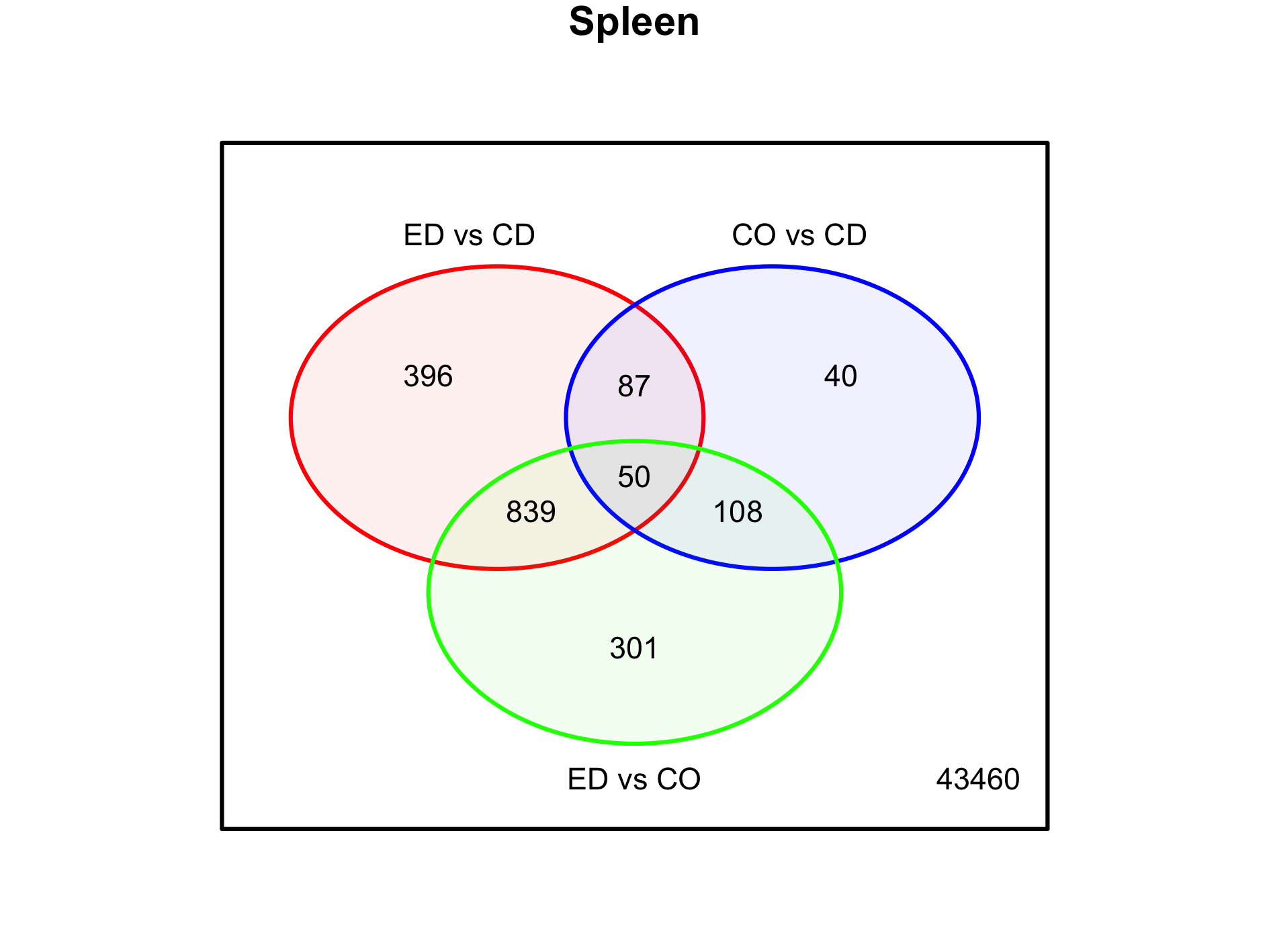 |
| --- |
| (**a**) |
| 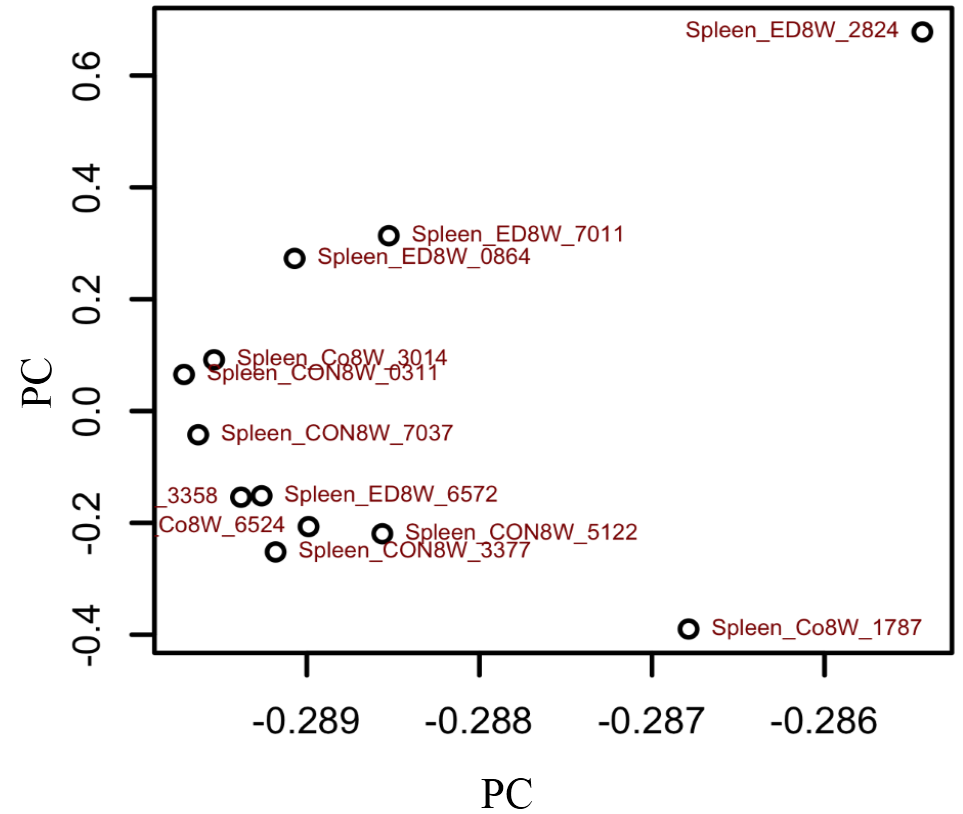 |
| (**b**) |
|  |
| (**c**) |
|  |
| (**d**) |

**Figure S1.** (**a**) Venn diagram depicting overlaps among differentially expressed genes upon dietary intervention. Most regulation can be seen for comparison HFD-ED vs. control diet (ED vs. CD), and HFD-ED vs. HFD-corn oil (ED vs. CO). HFD-corn oil vs. control diet (CO vs. CD) shows comparatively less bidirectional differentially regulated genes; (**b**) Principle Component Analysis (PCA) plot based on the normalized gene expression from the spleen tissue fed control, HFD-ED and HFD-corn oil is plotted for assessing the quality of the datasets. No animals or any related data was excluded from further assessment; (**c**) A heatmap showing significantly regulated BPs (*p*-value < 10 × 10^9^) without immune system process for every diet comparison after 8 weeks. Changes in the diet, especially HFD-ED positively regulate BPs mostly related to DNA repair and cell cycle down-regulates NF-κB, interleukin-12 and interferon-gamma related processes. HFD-ED = HFD-ED vs. control diet; HFD-CO = HFD-corn oil vs. control diet; ED-CO = HFD-ED vs. HFD-corn oil; (**d**) Illustration of linoleic acid and arachidonic acid signaling pathway analysis of the splenic transcriptome for the comparison HFD-ED vs. HFD-CO fed mice. The genes highlighted in red including *Gpx1* (glutathione peroxidase 1) and *Alox12* (arachidonate 12-lipoxigenase) are up-regulated and the genes in green including *Pla2g1b* (phospholipase A2 group IB), *Cyp4f14* (cytochrome P450 family 4 subfamily f polypeptide 14) and *Ptgis* (prostaglandin 12 synthase) are down-regulated. In black are the lipid mediators and synthetic intermediates including prostaglandins, prostacyclins, leukotrienes, 5-oxo-eicosatetraenoic acid, 15-oxo-eicosatetraenoic acid, 12-hydroxy-eicosatetraenoic acid, 20-hydroxy-eicosatetraenoic acid.
